# Comparative pharmacokinetics and bioavailability of albendazole sulfoxide in sheep and goats, and dose-dependent plasma disposition in goats

**DOI:** 10.1186/s12917-015-0442-5

**Published:** 2015-05-27

**Authors:** Dilek Aksit, Hande Sultan Yalinkilinc, Selim Sekkin, Murat Boyacioğlu, Veli Yilgor Cirak, Erol Ayaz, Cengiz Gokbulut

**Affiliations:** Department of Pharmacology and Toxicology, Faculty of Veterinary Medicine, Balikesir University, Balikesir, Turkey; Department of Pharmacology and Toxicology, Faculty of Veterinary Medicine, University of Adnan Menderes, Aydin, Turkey; Department of Parasitology, Faculty of Veterinary Medicine, University of Uludag, Bursa, Turkey; Department of Parasitology, Faculty of Medicine, Abant Izzet Baysal University, Bolu, Turkey; Department of Pharmacology, Faculty of Medicine, Balikesir University, Balikesir, Turkey

**Keywords:** Benzimidazoles, Albendazole, Albendazole Sulfoxide, Pharmacokinetics, Enantiomers, Sheep, Goat

## Abstract

**Background:**

The aims of this study were to compare the pharmacokinetics of albendazole sulfoxide (ABZ-SO, ricobendazole) in goats and sheep at a dose of 5 mg/kg bodyweight (BW), after intravenous (IV) and subcutaneous (SC) administrations, and to investigate the effects of increased doses (10 and 15 mg/kg BW) on the plasma disposition of ABZ-SO in goats following SC administration. A total of 16 goats (*Capra aegagrus hircus*, eight males and eight females) and 8 sheep (*Ovis aries*, four males and four females) 12–16 months old and weighing 20–32 kg, were used. The study was designed according to two-phase crossover study protocol. In Phase-1, eight sheep were assigned as Group I and 16 goats were allocated into two groups (Group II and Group III). ABZ-SO was applied to Group I (sheep) and Group II (goats) animals subcutaneously, and to Group III (goats) animals intravenously, all at a dose rate of 5 mg/kg BW. In Phase-2, the sheep in the Group I received ABZ-SO intravenously in a dose of 5 mg/kg BW; the goats in Group II and Group III received ABZ-SO subcutaneously at a dose of 10 mg/kg and 15 mg/kg BW, respectively. Blood samples were collected from the jugular vein at different times between 1 and 120 h after drug administrations. The plasma concentrations of ABZ-SO and its metabolites were analysed by high performance liquid chromatography.

**Results:**

In goats, the area under the curve, terminal half-life and plasma persistence of ABZ-SO were significantly smaller and shorter, respectively, compared with those observed in sheep following both IV and SC administrations at a dose of 5 mg/kg BW. On the other side, dose-dependent plasma dispositions of ABZ-SO were observed following SC administration at increased doses (10 and 15 mg/kg) in goats.

**Conclusions:**

Consequently, ABZ-SO might be used at higher doses to provide higher plasma concentration and thus to achieve greater efficacy against the target parasites.

## Background

Benzimidazole (BZD) and pro-BZD drugs are used widely to treat gastrointestinal helminthiasis including migrating larvae, liver flukes and lungworm infections in animals with a broad spectrum of activity and low mammalian toxicity [1]. The parent molecules of BZD anthelmintics are extensively metabolised in all animal species and the parent drug is short-lived and metabolic products predominate in systemic circulation. The primary metabolites, usually produced by oxidation and hydrolysis, are all more polar and water soluble than the parent drug. The poor water solubility reduces flexibility for drug formulation of the most potent BZD methylcarbamate anthelmintics such as albendazole (ABZ) and fenbendazole (FBZ), allowing their formulation only as tablets, boluses or suspensions for *per os*/intraruminal administration in ruminants [1]. After absorption from the intestine in ruminants, ABZ is rapidly metabolized into its anthelmintically active albendazole sulfoxide (ABZ-SO) and inactive albendazole sulfone (ABZ-SO_2_) metabolites by liver enzymes [2].

ABZ-SO, known as ricobendazole, is chemically the sulfoxide derivative of ABZ being the most important antelmintically active metabolic product found systematically after ABZ treatment in sheep [3–6] and cattle [7, 8]. ABZ-SO is much more water soluble compared with ABZ. An injectable formulation of ABZ-SO has been developed for subcutaneous (SC) administration in cattle and sheep. This formulation has some advantages compared with the other formulations for *per os* or intraruminal administration, as drug molecules are potentially freely available for absorption from the injection site, avoiding the first-pass effect and actions of the oesophageal groove [8]. The gastrointestinal and the first-pass metabolism are common metabolic pathways for sulfoxide BZDs and they are metabolised into their sulfoxides, which in turn are oxidized into the more polar and less anthelmintically active sulfone metabolites following *per os* administration in different animal species.

Sulfoxide BZDs [ABZ-SO and oxfendazole (OFZ)] which have a chiral centre about the sulfur atom are formed as metabolites of sulfides and are metabolised into sulfones. Pharmacodynamic and pharmacokinetic properties of enantiospecific pairs are commonly different and are of major importance for their effective and safe therapeutic use. The sulfones are anthelmintically inactive, whereas sulfides and sulfoxides are both active [9]. Although the plasma dispositions of two enantiomers of ABZ-SO and OFZ have been investigated in many species after *per os* administration of the pro-chiral ABZ, FBZ and racemic ABZ-SO and OFZ [10–19], there is a paucity of data available in the literature on the stereospecific plasma behaviour of the enantiomers of ABZ-SO following intravenous (IV) and SC administration of rac-ABZ-SO in goats.

Due to a shortage of registered drugs available for goats in most countries, different classes of drugs, including anthelmintics licensed for sheep are extensively used in goats without optimization of dosing regimens and determination of pharmacokinetic and pharmacodynamic properties [20]. It is generally acknowledged that the plasma disposition and metabolism of anthelmintic drugs are different between sheep and goats [21–25]. Goats metabolise and eliminate anthelmintic compounds more rapidly from blood compared with sheep. The presence of the metabolic differences between two species has been not considered for many years. The high prevalence of anthelmintic-resistant nematodes in goats are probably due to the extensive extra-label use of these compounds at a standard ovine dosage, corresponding to a drug under-dosing and leading to reduced efficacy of the drug [26, 20]. Therefore, the present study was designed to compare the pharmacokinetic and bioavailability of ABZ-SO in goats and sheep following IV and SC administrations at a dose rate of 5 mg/kg bodyweight (BW) and to investigate the effects of increased doses (10 and 15 mg/kg) on the plasma disposition of ABZ-SO in goats following SC administration. In addition, the stereospecific disposition of enantiomers [(+) ABZ-SO and (−) ABZ-SO)] was also determined and compared in both species after IV and SC administrations of racemic (rac)-ABZ-SO.

## Results

**Fig. 1:**
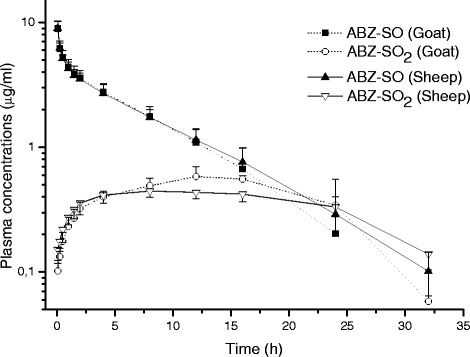
Comparative mean (±SD) plasma concentration *vs.* time curves of ABZ-SO and ABZ-SO_2_ in goats and sheep following intravenous administrations at a dose of 5 mg/kg (n = 8)

The analytical procedures for the determination of plasma concentrations of ABZ, albendazole-2-aminosulfone (ABZ-NH_3_), ABZ-SO and ABZ-SO_2_ were validated before analysing of the experimental samples and the validation parameters for all molecules are summarised in Table 1. ABZ and ABZ-NH_3_ were not detected in any plasma samples of sheep and goats following either IV or SC administrations. The plasma concentration *vs*. time curves of ABZ-SO and ABZ-SO_2_ are shown in Fig. 1 and the pharmacokinetic data are summarized in Table 2 following IV administration in goats and sheep. Although the absorption phase and peak plasma concentrations (C_max_) of ABZ-SO were similar in both species, the area under the curve (AUC), terminal half-life (T_1/2_) and plasma persistence (MRT) values were smaller and shorter, respectively, in goats compared with those observed in sheep after SC administration at a dose of 5 mg/kg BW.

**Table 1:** Validation of analytical method used for determination of ABZ, ABZ-NH_3_, ABZ-SO and ABZ-SO_2_ concentrations in plasma

	ABZ-NH_3_	ABZ-SO	ABZ-SO_2_	ABZ
LOD (μg/ml)	0.015	0.016	0.016	0.017
LOQ (μg/ml)	0.042	0.045	0.045	0.050
Range of linearity (μg/ml)	0.05-20	0.05-20	0.05-20	0.05-20
Linearity (*r* ^*2*^)	0.997	0.999	0.998	0.996
Recovery (%)	63.69 (5.42)	94.10 (6.39)	91.90 (4.88)	69.29 (6.21)
Coefficient of variation (%)	6.89	5.35	6.33	7.75
Retention times (min)	5.37	5.77	6.81	9.95

*LOD* limit of detection, *LOQ* limit of quantification. Values in the brackets represent the coefficient of variations for the recovery assays, *r*: correlation coefficient

**Table 2:** Comparative mean (±SD) kinetic parameters of ABZ–SO and ABZ–SO_2_ in goats and sheep following intravenous administrations at a dose of 5 mg/kg (n = 8)

Parameters	Goat		Sheep	
	ABZ-SO	ABZ-SO_2_	ABZ-SO	ABZ-SO_2_
T_1/2λz_ (h)	5.43 ± 0.89*	3.53 ± 0.81*	6.57 ± 0.73	11.43 ± 9.41
C_max_ (μg/mL)	-	0.62 ± 0.21	-	0.46 ± 0.05
C_0_ (μg/mL)	10.84 ± 1.81	-	10.86 ± 1.82	-
T_max_ (h)	-	15.00 ± 6.48*	-	8.50 ± 3.34
T_last_ (h)	21.00 ± 4.14*	25.00 ± 10.56	30.00 ± 3.70	31.00 ± 2.83
AUC_last_ (μg.h/mL)	34.10 ± 5.31*	10.49 ± 4.26	39.15 ± 5.59	11.00 ± 0.78
AUCM_last_ (μg.h^2^/mL)	201.91 ± 58.84*	133.34 ± 65.25	293.46 ± 64.66	155.96 ± 17.30
Cl (L/h/kg)	0.14 ± 0.03	-	0.13 ± 0.02	-
MRT_last_ (h)	5.81 ± 0.86*	12.31 ± 4.95	7.44 ± 1.03	14.18 ± 1.28
Vd_ss_ (L/kg)	0.94 ± 0.09	-	1.04 ± 0.14	-

*T*_*1/2λz*_ terminal half-life; *C*_*max*_ peak plasma concentration; *C*_*0*_ plasma concentration at time 0; *T*_*max*_ time to reach peak plasma concentration; *T*_*last*_ time to last detectable plasma concentration; *AUC*_*last*_ area under the (zero moment) curve from time 0 to the last detectable concentration; *AUCM* area under the moment curve from time 0 to t last detectable concentration; *Cl* clearance of drug; *MRT*_*last*_ mean residence time; *Vd*_*ss*_ volume of distribution at steady-state

*The parameters in goats are significantly different from those in sheep (*P < 0.05)

**Fig. 2:**
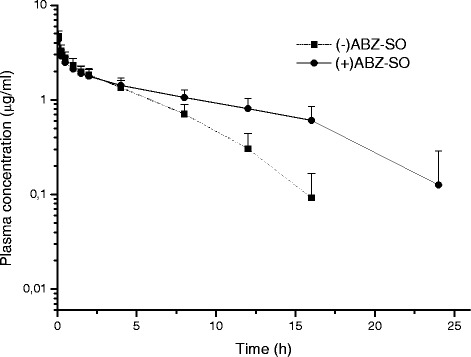
Mean (±SD) plasma concentrations of enantiomers [(+)ABZ-SO and (−)ABZ-SO)] vs. time curves of ABZ-SO in goats following intravenous administrations at a dose of 5 mg/kg (n = 8)

**Fig. 3:**
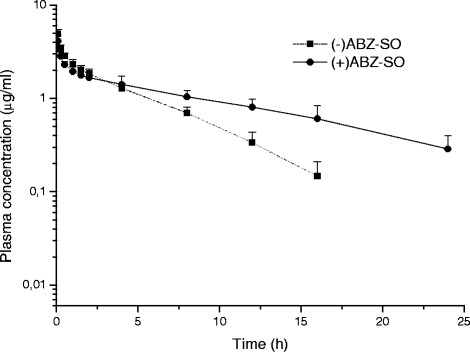
Mean (±SD) plasma concentrations of enantiomers [(+)ABZ-SO and (−)ABZ-SO)] vs. time curves of ABZ-SO in sheep following intravenous administrations at a dose of 5 mg/kg (n = 8)

**Fig. 4:**
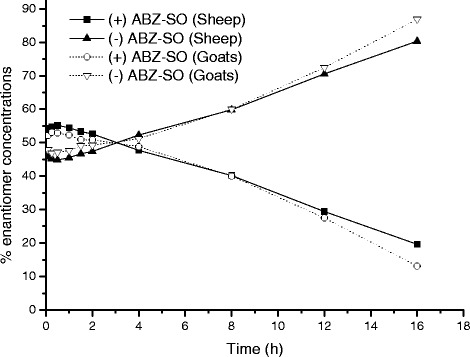
Comparative ratio of the percentage of enantiomers (ABZ-SO and (−) ABZ-SO) vs. time curves of ABZ-SO in goats and sheep following intravenous administrations at a dose of 5 mg/kg (n = 8)

The plasma concentrations of (+) ABZ-SO and (−) ABZ-SO) *vs*. time curves of ABZ-SO in goats and sheep following IV administrations at a dose rate of 5 mg/kg BW are shown in Fig. 2 and 3, respectively. In addition, the comparative ratio of the percentage of enantiomers in goats and sheep following IV administrations is shown in Fig. 4 and kinetic parameters of each enantiomer are summarised in Table 3. Stereospecific disposition of enantiomers displayed similar disposition in sheep and goats. (+) ABZ-SO were predominant and displayed significantly higher plasma concentrations compared with (−) enantiomer. The AUC of (+) enantiomer was almost two times larger than that of (−) enantiomer in both species after IV administration of rac-ABZ-SO.

**Table 3:** Comparative mean (±SD) kinetic parameters of (−) ABZ-SO and (+) ABZ-SO in goats and sheep following intravenous administrations at a dose of 5 mg/kg (n = 8)

Parameters	Goat		Sheep	
	(−) ABZ-SO	(+) ABZ-SO	(−) ABZ-SO	(+) ABZ-SO
T_1/2λz_ (h)	3.66 ± 0.66*	8.03 ± 1.67	3.98 ± 0.64*	9.36 ± 2.23
C_max_ (μg/mL)	2.32 ± 0.40	2.11 ± 0.33	2.33 ± 0.27	1.95 ± 0.29
T_last_ (h)	15.43 ± 1.51*	20.57 ± 4.28	17.00 ± 2.83*	30.00 ± 3.70
AUC_last_ (μg.h/mL)	11.73 ± 2.43*	18.45 ± 5.62	11.96 ± 1.50*	20.53 ± 3.90
AUCM_last_ (μg.s^2^/mL)	51.33 ± 16.00	135.35 ± 62.98	55.53 ± 9.48*	186.82 ± 49.46
Cl (L/h/kg)	0.42 ± 0.10*	0.24 ± 0.06	0.40 ± 0.06	0.24 ± 0.06
MRT_last_ (h)	4.29 ± 0.57*	7.07 ± 1.20	4.63 ± 0.38*	8.98 ± 1.44
Vd_ss_ (L/kg)	2.05 ± 0.24	2.53 ± 0.41	2.23 ± 0.27	2.47 ± 0.30

*T*_*1/2λz*_ terminal half-life; *C*_*max*_ peak plasma concentration; *T*_*last*_ time to last detectable plasma concentration; *AUC*_*last*_ area under the (zero moment) curve from time 0 to the last detectable concentration; *AUCM* area under the moment curve from time 0 to t last detectable concentration; *Cl* clearance of drug; *MRT*_*last*_ mean residence time; Vd_ss_: volume of distribution at steady-state

*Differences between the enantiomers in the same animal species (P < 0.05)

**Fig. 5:**
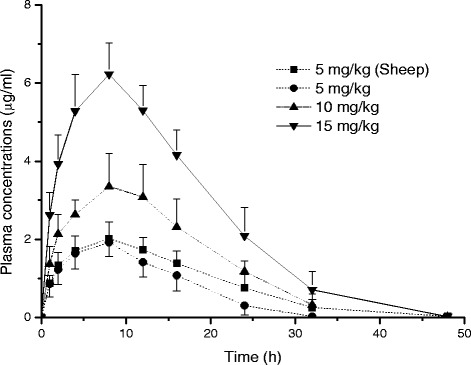
Comparative mean (±SD) plasma concentration *vs*. time curves of ABZ-SO following subcutaneous administrations in sheep and goats at a dose rate of 5 mg/kg and at increased dose rates (10 and 15 mg/kg) in goats (n = 8)

**Fig. 6:**
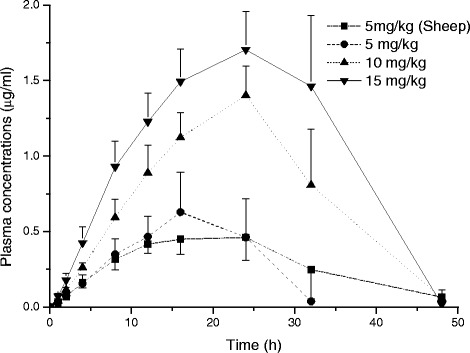
Comparative mean (±SD) plasma concentration vs. time curves of ABZ-SO_2_ following subcutaneous administrations of ABZ-SO in sheep and goats at a dose of 5 mg/kg and at increased dose rates (10 and 15 mg/kg) in goats (n = 8)

**Fig. 7:**
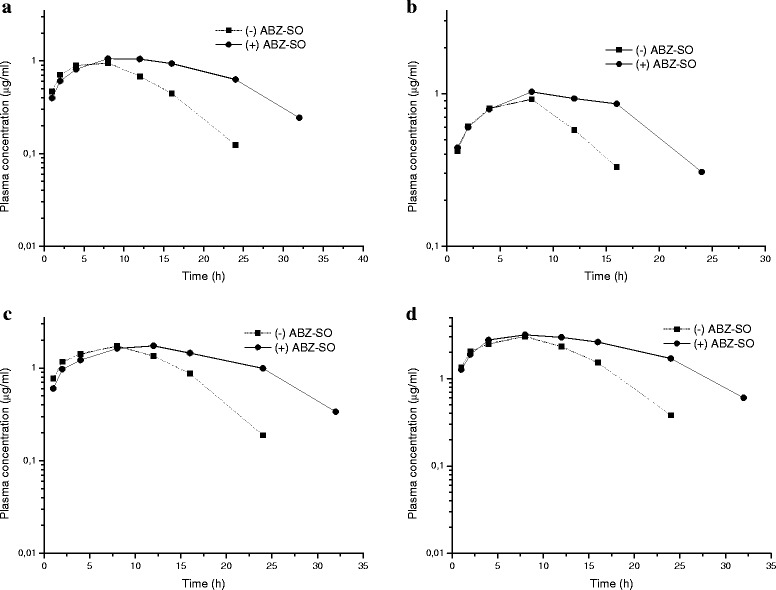
Mean plasma concentrations of enantiomers [(+) ABZ-SO and (−) ABZ-SO)] *vs*. time curves of ABZ-SO in sheep (5 mg/kg-Fig. **a**) and goats following subcutaneous administrations at doses of 5 (Fig. **b**), 10 (Fig. **c**) and 15 (Fig. **d**) mg/kg (n = 8)

The plasma concentration *vs*. time curves of ABZ-SO and ABZ-SO_2_ are shown in Fig. 5 and 6, respectively. Mean (±SD) pharmacokinetic parameters of ABZ-SO and its metabolite ABZ-SO_2_ in goats and sheep at a dose rate of 5 mg/kg and at increased doses (10 and 15 mg/kg BW) in goats following SC administrations are summarized in Table 4 and 5, respectively. Dose-dependent plasma dispositions of ABZ-SO were observed following SC administration at increased doses (10 and 15 mg/kg BW) in goats. In addition, the mean plasma concentrations of enantiomers *vs*. time curves of ABZ-SO are shown in Fig. 7. The mean kinetic parameters of both enantiomers in goats and sheep at a dose of 5 mg/kg BW and at increased dose rates (10 and 15 mg/kg BW) in goats following SC administrations are given in Table 6.

**Table 4:** Comparative mean (±SD) kinetic parameters of ABZ-SO in goats and sheep at a dose rate of 5 mg/kg and at increased dose rates (10 and 15 mg/kg) in goats following subcutaneous administrations (n = 8)

Parameters	Sheep	Goats		
	5 mg/kg	5 mg/kg	10 mg/kg	15 mg/kg
T_1/2λz_ (h)	4.99 ± 1.12*	2.44 ± 0.37^b^	4.62 ± 1.51	5.79 ± 1.57
T_max_ (h)	8.50 ± 1.41	7.43 ± 1.51	8.00 ± 2.31	7.50 ± 1.41
C_max_ (μg/mL)	2.05 ± 0.37	1.98 ± 0.37^b^	3.40 ± 0.84^c^	6.30 ± 0.81
T_last_ (h)	38.00 ± 8.28*	28.57 ± 4.28	36.57 ± 7.81	35.00 ± 8.49
AUC_last_ (μg.h/mL)	38.35 ± 9.81*	29.76 ± 6.52^b^	62.19 ± 13.61^c^	112.66 ± 17.16
MAT (h)	5.56 ± 2.07	4.53 ± 2.23^b^	7.07 ± 1.49	7.10 ± 1.30
MRT_last_ (h)	13.00 ± 1.54*	10.34 ± 1.85	12.88 ± 1.19	12.50 ± 1.70
C_max_ (μg/mL) (Normalized)^a^	2.05 ± 0.37	1.98 ± 0.37	1.70 ± 0.42	2.10 ± 0.27
AUC (μg.h/mL) (Normalized)^a^	38.35 ± 9.81	29.76 ± 6.52	31.09 ± 6.30	37.55 ± 5.72
*F* (%)	97.9*	82.0^b^	91.18^c^	110.01

*T*_*1/2λz*_ terminal half-life; *T*_*max*_ time to reach peak plasma concentration; *C*_*max*_ peak plasma concentration; *T*_*last*_ time to last detectable plasma concentration; *AUC*_*last*_ area under the (zero moment) curve from time 0 to the last detectable concentration; *MRT*_*last*_ mean residence time; *MAT* mean absorption time; *F* bioavailability

*The parameters in sheep are significantly different from those in goats (*P < 0.05)

^a^AUC and C_max_ values were dose-normalized dividing the observed value by 2 (10 mg/kg) or 3 (15 mg/kg)

^b^The parameters in goats observed after a dose of 5 mg/kg are significantly different from those in goats observed after doses of 10 and 15 mg/kg (&P < 0.05)

^c^The parameters in goats observed after a dose of 10 mg/kg are significantly different from those in goats observed after a dose of 15 mg/kg (&P < 0.05)

**Table 5:** Comparative mean (±SD) kinetic parameters of ABZ-SO_2_ in goats and sheep following subcutaneous administrations of ABZ-SO at a dose rate of 5 mg/kg and at increased dose rates (10 and 15 mg/kg) in goats (n = 8)

Parameters	Sheep	Goats		
	5 mg/kg	5 mg/kg	10 mg/kg	15 mg/kg
T_1/2λz_ (h)	4.57 ± 2.36*	1.92 ± 0.37	2.72 ± 0.88	2.43 ± 0.47
T_max_ (h)	18.50 ± 4.75	17.14 ± 5.52^&^	25.14 ± 3.02	26.00 ± 5.66
C_max_ (μg/mL)	0.53 ± 0.10	0.68 ± 0.25^&^	1.41 ± 0.20	1.75 ± 0.26
T_last_ (h)	42.00 ± 8.28*	30.86 ± 3.02	41.14 ± 8.55	46.00 ± 5.66
AUC_last_ (μg.h/mL)	12.34 ± 3.82	11.43 ± 3.20^&^	30.52 ± 6.84^¥^	41.96 ± 6.55
MRT_last_ (h)	19.78 ± 3.42	16.16 ± 2.05	20.74 ± 3.02	20.79 ± 2.23
C_max_ (μg/mL) (Normalized)^#^	0.53 ± 0.10	0.68 ± 0.25	0.70 ± 0.10	0.58 ± 0.08
AUC (μg.h/mL) (Normalized)^#^	12.34 ± 3.82	11.43 ± 3.20	15.26 ± 3.42	13.98 ± 2.18

*T*_*1/2λz*_ terminal half-life; *T*_*max*_ time to reach peak plasma concentration; *C*_*max*_ peak plasma concentration; *T*_*last*_ time to last detectable plasma concentration; *AUC*_*last*_ area under the (zero moment) curve from time 0 to the last detectable concentration; *MRT*_*last*_ mean residence time

*The parameters in sheep are significantly different from those in goats (*P < 0.05)

^#^AUC and C_max_ values were dose-normalized dividing the observed value by 2 (10 mg/kg) or 3 (15 mg/kg)

^&^The parameters in goats observed after a dose of 5 mg/kg are significantly different from those in goats observed after doses of 10 and 15 mg/kg (&P < 0.05)

^¥^The parameters in goats observed after a dose of 10 mg/kg are significantly different from those in goats observed after a dose of 15 mg/kg (&P < 0.05)

**Table 6:** Comparative mean (±SD) kinetic parameters of (−) ABZ-SO and (+) ABZ-SO in goats and sheep following subcutaneous administrations at dose rates of 5, 10 and 15 mg/kg (n = 8)

Parameters	Sheep		Goats					
	5 mg/kg		5 mg/kg		10 mg/kg		15 mg/kg	
	(−) ABZ-SO	(+) ABZ-SO	(−) ABZ-SO	(+) ABZ-SO	(−) ABZ-SO	(+) ABZ-SO	(−) ABZ-SO	(+) ABZ-SO
T_1/2λz_ (h)	6.23 ± 1.71	9.74 ± 6.40	4.56 ± 1.55	7.95 ± 4.59	4.79 ± 1.57	7.36 ± 3.51	4.85 ± 1.93^*^	7.57 ± 3.29
T_max_ (h)	7.43 ± 1.51	9.14 ± 1.95	8.00 ± 0.00	8.80 ± 1.79	7.43 ± 1.51	10.29 ± 3.15	8.00 ± 0.00	8.00 ± 2.14
C_max_ (μg/mL)	0.95 ± 0.22	1.12 ± 0.21	0.92 ± 0.24	1.03 ± 0.20	1.74 ± 0.45	1.75 ± 0.46	3.05 ± 0.33	3.38 ± 0.84
AUC_last_ (μg.h/mL)	15.27 ± 2.67^*^	28.22 ± 10.36	12.48 ± 4.14^*^	21.99 ± 6.96	25.41 ± 4.93*	40.00 ± 9.03	44.12 ± 6.84^*^	74.73 ± 16.43
MRT_last_ (h)	12.20 ± 1.94^*^	19.45 ± 7.91	10.20 ± 1.60	16.15 ± 6.43	10.82 ± 1.26*	16.74 ± 3.27	10.34 ± 1.14^*^	16.49 ± 3.32

*T*_*1/2λz*_ terminal half-life; *T*_*max*_ time to reach peak plasma concentration; *C*_*max*_ peak plasma concentration; *AUC*_*last*_ area under the (zero moment) curve from time 0 to the last detectable concentration; *MRT*_*last*_ mean residence time

*Differences between the enantiomers in the same animals and dose rates (P < 0.05)

## Discussions

The present study showed that the AUC and MRT values of ABZ-SO in goats were significantly smaller and shorter compared with those observed in sheep. Moreover, T_1/2_ of ABZ-SO was significantly shorter in goats compared with that observed in sheep following IV and SC administrations at a dose of 5 mg/kg BW. The origin of the lower plasma concentration in goats is unclear. The most likely explanation for the origin of these kinetic differences is that goats have a greater metabolic capacity and elimination capability of ABZ-SO in comparison with sheep. Previous studies indicated that the plasma disposition and metabolism of anthelmintic drugs are different between sheep and goats [21–25]. It has been commonly acknowledged that the anthelmintic drugs are more rapidly metabolised and eliminated from blood in goats compared with sheep. This difference has been shown for different anthelmintic compounds, including BZDs [19, 21, 27–29] endectocides [24, 30, 31], levamisole, [25, 32] and oxyclozanide [25]. The greater ability to detoxify exogenous compounds, including anthelmintics, has been attributed to the specific feeding behaviour of goats [33], since the feeding behaviour of goats is quite different compared with that of sheep. Sheep are known as grazers, preferring to feed on grass and forbs, whereas goats are described as ingesting substantial amounts of browse (woody plants, vines and brush). Thus, goats are better adapted to tolerate and detoxify plant toxins and exogenous compounds compared with sheep [34, 35].

The plasma disposition of ABZ-SO has been previously reported in sheep after IV and SC administration at a dose of 5 mg/kg BW [8]. Some findings in the present study (T_1/2_: 5.57 h, AUC: 39.15 μg.h/mL, MRT: 7.44 h, Cl: 0.13 L/h/kg and Vd_ss_: 1.04 L/kg) are similar to those obtained by Formentini *et al*. [8] (T_1/2_: 5.19 h, AUC: 29.6 μg.h/mL, MRT: 6.4 h, Cl: 0.17 L/h/kg and Vd_ss_: 1.3 L/kg) in sheep following IV administration at a same dose rate (5 mg/kg BW). On the other hand, although C_max_ (2.05 μg/mL *vs*. 2.09 μg/mL), T_1/2_ (MRT (13.00 h *vs*. 10.4 h) and F (97.9 % vs. 96.0 %) values of ABZ-SO are similar, the AUC (38.35 μg.h/mL vs. 28.4 μg.h/mL) T_max_ (8.5 h vs. 3.81 h), and MAT (5.56 h vs. 3.14 h) values are larger and longer than those reported by Formentini *et al*. [8] in sheep, respectively. The origin of the differences between the studies is unclear. These differences between the studies may be attributable to differences in methodology or experimental conditions such as different feeding type or regime, or even to parasitological status of the animals that may cause differences in absorption, disposition and persistence of anthelmintic drugs in the animals.

The results obtained in the present study indicate that an increase in ABZ-SO dosage in goats is associated with elevation in the plasma level of ABZ-SO. Significantly higher AUC and C_max_ values for ABZ-SO were observed after SC administration at both dose rates of 10 and 15 mg⁄ kg compared to the treatment at 5 mg⁄ kg (Table 2). The AUC of ABZ-SO increased from 29.76 (5 mg/kg) to 62.19 (10 mg/kg) and to 112.66 μg.h/mL (15 mg/kg). These findings are in agreement with the previous study performed by Alvarez *et al*. [36] who indicated that increasing the dose of ABZ (5, 15, 45 mg/kg BW) is associated with enhanced plasma level and exposure of ABZ metabolites in sheep after intraruminal administration.

The plasma dispositions of the two enantiomers of ABZ-SO have been investigated in many species after oral administration of the pro-chiral ABZ [10–12, 15–18, 29], and after IV and *per os* administration of ABZ-SO in sheep [5, 17] which is discussed by Capece *et al*. [37]. In addition, it has been shown that (+) ABZ-SO was anthelmintically more potent than rac-ABZ-SO and (−) ABZ-SO by using an *ex vivo* murine model for *Trichinella spiralis* infection [38].

The current study also showed that the enantiomers of ABZ-SO were never in racemic proportions and the enantiospecific ratio (+/−) of plasma concentration of ABZ-SO changed over time in favour of the (+) enantiomer in sheep and goats after both IV and SC administration of rac-ABZ-SO. The AUC of the (+) enantiomer was almost 2 times larger than that of (−) enantiomer of ABZ-SO, in agreement with the previous studies performed by Capece *et al*. [5] who indicated that (+) enantiomer represented 85 and 80 % of the total plasma AUC of ABZ-SO in male and female sheep, respectively. This may contribute to the anthelmintic effect of the (+) ABZ-SO enantiomer since it has been shown that (−) ABZ-SO is quickly metabolised into the inactive sulfone [17]. It has been demonstrated that the flavin monooxygenase (FMO) system is enantioselective in favour of the (+) sulfoxide of ABZ, whereas only cytochrome P450 systems specifically produce (−) ABZ-SO which was shown to be the main substrate for the formation of the inactive sulphone metabolite [11,39]. Differences in the interspecies enantioselectivity could be explained by different metabolic enzyme contributions.

## Conclusion

Although the absorption phase and the peak plasma concentration of ABZ-SO were similar in both species, the plasma availability, elimination and persistence of ABZ-SO were significantly lower and shorter in goats compared with those observed in sheep, respectively, following SC administrations at a dose of 5 mg/kg BW. As a consequence, treatment of goats with ABZ-SO at the recommended sheep dose may result in reduced anthelmintic efficacy, which may increase the risk of drug resistance in internal parasites. Moreover, it was also shown that increasing the dose of ABZ-SO in goats was associated with enhanced plasma exposures after SC administration. Therefore, increased doses could be a strategy to provide higher and more persistent plasma concentration and thus to improve the efficacy against the target parasites and to delay the development of anthelmintic resistance in goat parasites.

## Methods

### Experimental animals

A total of 16 goats (*Capra aegagrus hircus*, eight male and eight female) and 8 sheep (*Ovis aries*, four male and four female) 12–16 months old and weighing 20–32 kg, were used. The animals were housed and fed twice daily with an appropriate quantity of feed during the experiment period. Water was supplied *ad libitum*. This study was approved by the Animal Ethic Committee of University of Adnan Menderes. The animals were allocated into three groups (Groups I, Group II and Group III) of 8 such that the mean weight and sex of animals in each group was similar.

### Drug administration and sampling

The study was designed according to two-phase crossover study protocol. A four-week washout period was allowed between the phases of the study. In both phases, 8 sheep were assigned as Group I, and 16 goats were allocated into two groups (Group II and III). For treatment of the animals, ABZ-SO (ricobendazole; Rizal® injectable, 100 mg/mL, Sanovel, Istanbul, Turkey) was used.

In Phase 1, the treatments were as follows: Group I and II (both subcutaneously: 5 mg/kg BW-recommended sheep dose), and Group III (intravenously: 5 mg/kg BW). A four-week washout period was allowed between the phases of the study.

In Phase 2, following treatments were performed: Group I (intravenously: 5 mg/kg BW), Group II and III (both subcutaneously; 10 and 15 mg/kg BW, respectively). Because of the possible irritation at the injection site, each of these doses (10 mg/kg and 15 mg/kg) were divided into two injections and applied to left and right side of the goats.

Heparinized blood samples (5 ml) were collected via jugular venipuncture prior to drug administration (time 0) and 1, 2, 4, 8, 12, 16, 24, 32, 48, 72, 96, 120 h after SC administration. Additionally, 1, 5, 15, 30 and 90 min samples were collected after IV administrations in goats and sheep of intravenous groups. Blood samples were centrifuged at 2000 X g for 20 min, and plasma was harvested and transferred to plastic tubes. All plasma samples were stored at−20 °C until the analyses.

### Analytical procedures

Pure analytical standard compounds of ABZ, ABZ-NH_3_, rac-ABZ-SO, ABZ-SO_2_ and internal standard of oxibendazole (OBZ) were obtained from Dr. Ehrenstorfer (Augsburg, Germany). A stock solution (100 μg/mL) of a pure standard mixture was prepared with acetonitrile as the solvent. This was diluted with acetonitrile-water (25:75, v/v) to give 0.5, 1, 5, 10 and 20, 50 standard solutions for calibration as standard curves and to add to the drug-free plasma samples to determine the recovery.

Plasma concentrations of ABZ, ABZ-NH_3_, ABZ-SO and ABZ-SO_2_ were estimated by high performance liquid chromatography (HPLC) with a liquid-liquid phase extraction procedure adapted from that described by Marriner and Bogan [3]. Briefly, drug-free plasma samples (1 ml) were spiked with standards of ABZ, ABZ-NH_3_, rac-ABZ-SO and ABZ-SO_2_ to reach the following final concentrations: 0.05, 0.1, 0.5, 1, 2, 5 and 10 μg/mL. OBZ (0.5 μg/mL) was used as an internal standard. Ammonium hydroxide (100 μl, 0.1 N, pH 10) was added to 10 ml-ground glass tubes containing 1 mL spiked or experimental plasma samples. After mixing for 15 s, 6 mL ethyl acetate was added. The sample tubes were stoppered and shaken for 10 min on a slow rotary mixer. After centrifugation at 3000 g for 10 min, the upper organic phase (4 ml) was transferred to a thin-walled 10 ml-conical glass tube and evaporated to dryness at 40 °C in a rota vapour (Maxi-Dry plus, Heto, Denmark).

The dry residue was reconstituted with 250 μl mobile phase. Then the tubes were placed in an ultrasonic bath and finally, 50 μl of this solution was injected into the chromatographic system.

### Chromatographic conditions

The mobile phase was a mixture of acetonitrile-water to which glacial acetic acid was added (0.5 %, v/v). It was pumped through the column (Luna nucleosil C_18_, 3 μm, 150 mm × 4.6 mm Phenomenex, Cheshire, UK) with nucleosil C_18_ guard column (Phenomenex, Cheshire, UK) in a linear gradient fashion changing from 10:90 (acetonitrile-water) to 85:15 for 11 min; 85:15 to 10:90 for 1 min and the last ratio was maintained for 5 min. The flow rate was 1 ml/min. Samples were processed on a computerized gradient HPLC system (1100 series, Agilent Technologies, GmbH, Germany) comprising a degasser, a quaternary pump (G1354A), an auto sampler (G1313), a column oven (G1316A) and diode-array detector (G1315B) set at 292 nm for all molecules.

The extracted samples were re-analysed by a chiral stationary phase to determine the concentration of ABZ-SO enantiomers. The enantiomers were estimated by using chiral chromatography adapted from that previously described by Delatour *et al*. [11] with some modifications. Briefly, a mobile phase of acetonitrile:water (7:93) was pumped at a flow rate of 1 ml/min through a Chiral-AGP column (5 μm, 150 × 40 mm, ChromTech, MN, USA) with ultraviolet detection at 292 nm for 6 min and then the mobile phase ratio was changed to 100 % acetonitrile and maintained for 4 min to wash column for less polar molecules and impurities and finally the ratio changed to initial proportion and (7:93) maintained for 3 min to prepare for the next injection.

### Method of calibration

The analytic methods used for ABZ, ABZ-NH_3_, rac-ABZ-SO and ABZ-SO_2_ in plasma were validated prior to the start of the study. The analyte was identified with the retention times of the pure reference standards. Recoveries of the analytes were measured by comparison of the peak areas from 7 spiked plasma samples with the areas resulting from injection of external and internal standards. The inter-and inra-assay precisions of the extraction and chromatography procedures was evaluated by processing replicate aliquots of drug-free sheep and goat plasma samples containing known amounts of the drugs on different days.

The limits of detection (LOD) and quantification (LOQ) were determined based on signal to noise ratios of 3 and 10, respectively, taken by measuring the instability of the baseline before and after each molecule signal, using individual injections. Calibration curves were fitted by use of 7 concentrations that ranged from 0.05 to 10 μg/mL for plasma samples.

### Pharmacokinetics and statistical analysis of data

The plasma concentration versus time curves obtained after each treatment in individual animals were fitted with a software program (WinNonlin, version 5.2, Pharsight Corp, Mountain View, California) and reported as mean ± SD. The pharmacokinetic parameters for each animal were analysed via non-compartmental model analysis for both administration routes. The C_max_ and T_max_ were obtained from the plotted plasma concentration-time curve in each animal. The trapezoidal rule was used to calculate the area under the curve (AUC), and mean residence time (MRT) from 0 to last time with a measurable concentration was calculated by use of the following equation:

$$ \mathrm{M}\mathrm{R}{\mathrm{T}}_{\mathrm{last}} = \mathrm{A}\mathrm{U}\mathrm{M}{\mathrm{C}}_{\mathrm{last}}/\mathrm{AU}{\mathrm{C}}_{\mathrm{last}} $$

Where AUMC_last_ is the area under the moment curve from 0 to infinity and AUC_last_ is the AUC from 0 to infinity.

Terminal half-life (T_1/2λz_) was calculated as:

$$ {\mathrm{T}}_{1/2\uplambda \mathrm{z}}=\hbox{--} \ln (2)/{\uplambda}_{\mathrm{z}} $$

Where λz represent the first order rate constant associated with the terminal (log linear) portion of the curve. The mean absorption time (MAT) was calculated by the following equations:

$$ \mathrm{M}\mathrm{AT} = \mathrm{M}\mathrm{R}{\mathrm{T}}_{SC}\hbox{--} \mathrm{M}\mathrm{R}{\mathrm{T}}_{\mathrm{IV}} $$

The fraction of dose absorbed (ie, *F*) was calculated by use of mean AUCs calculated for each route of administration by use of the following equation:

$$ F = \left(\mathrm{AU}{\mathrm{C}}_{SC} \times {\mathrm{D}}_{IV}/\ \mathrm{AU}{\mathrm{C}}_{IV} \times {\mathrm{D}}_{SC}\right) \times 100 $$

The pharmacokinetic parameters are reported as mean ± SD. Pharmacokinetic parameters were statistically compared with a one-way analysis of variance (ANOVA). All statistical analyses were performed by using MINITAB for Windows (release 12.1, Minitab Inc., State College, PA, USA). Mean values were considered significantly different at *P* < 0.05.

## Abbreviations

ABZ: Albendazole
ABZ-SO: Albendazole sulfoxide
ABZ-SO_2_: Albendazole sulfone
ABZ-NH_3_: Albendazole-2-aminosulfone
BZD: Benzimidazole
Pro-BZDs: Pro-benzimidazoles
FBZ: Fenbendazole
HPLC: High performance liquid chromatography
T_1/2λz_: Terminal half-life
T_max_: Time to reach peak plasma concentration
C_max_: Peak plasma concentration
T_last_: Time to last detectable plasma concentration
AUC_last_: Area under the (zero moment) curve from time 0 to the last detectable concentration
MRTlast: Mean residence time
Cl: Clearance of drug
Vd_ss_: Volume of distribution at steady-state
